# Supplementary material for: AquaCropPlotter: A Shiny app for visualizing and analyzing AquaCrop simulation results
Source: PLoS One. 2025 Dec 2;20(12):e0337705. doi: 10.1371/journal.pone.0337705 (PMC12671777; doi:10.1371/journal.pone.0337705)
Supplement: Attachment — Submitted filename: _AquacropPlotter_PlosOne_response to reviewers.docx [file pone.0337705.s003.docx]

**Response to reviewers**

**Reviewer #1:** The authors have made a valuable contribution to the crop modeling and natural resource management community by developing a comprehensive set of tools for visualizing and analyzing AquaCrop outputs. These tools include a Windows GUI, a ShinyApp, and an R package. Additionally, the paper presents a case study conducted across three locations under different climate change scenarios. The manuscript is generally well-structured, clearly written, and follows a logical progression that is easy to follow. However, I have some concerns and suggestions that I provide detailed comments below.

- Comment 1: In the introduction, the authors present the importance and implications of developing an application for visualizing and analysing AquaCrop, which is well-done. However, the authors should bear in mind that there will be two kinds of readers: the ones that are acquainted with AquaCrop and understand all the equations and how the physical processes are represented in the model and the ones that will be firstly introduced by AquaCrop by this paper. While I understand that the main target group can potentially be the reader that is acquainted with AquaCrop, I think that giving more details of AquaCrop is of utmost importance. This includes, but it is not limited to, details of the input data that is required and the units (maybe a table?), the main equations and physical principles, and limitations of the model.

Response: We have added more information about AquaCrop including the principles, inputs, outputs, and limitations (Line 58 - 110). We feel that listing all the inputs and their units might be quite detailed for the purpose of this manuscript, since there are many inputs that are not straightforward and there can be multiple units for a single variable, e.g. for the climate inputs. We think that providing the concepts and summary of the required inputs may be sufficient for understanding AquaCrop within the scope of this manuscript. More details of inputs can be found better explained in detail in the original AquaCrop publications cited.

- Comment 2: The citation and reference of the packages used in to develop the R application is essential, however, it is important to explain what is the purpose of using a given package. The authors have done this by explaining that Tudyverse and furr were used for analysis pipelines. A similar explanation should be given for shinydashboard, shinyjs, shinyBS, and DT.

Response: We have added more explanation for the packages used (Line 158 - 164).

- Comment 3: The authors have done an excellent job in providing all the relevant links for accessing all the extensive resources related to AquaCropPlotter (github and Mendeley repositories, links for download and technical documentation). All links are working correctly, nonetheless, I strongly suggest presenting them as a citation and put the links in the reference.

Response: We have put the links in the references and cited them in the text (Line 166-178).

- Comment 4: Maybe one of the most critical aspects of AquaCropPlotter is the ingestion of the data into the application. As the authors stated "AquaCropPlotter takes project files and output files from AquaCrop simulation as its input". I think the authors should provide more information on the differences of the Standard "GUI" and the stand-alone "Plug-in", e.g., in what cases each one of them are used? Also, the output files from each type of interface should be better explained. The standard GUI saves 10 files and the reader should learn in the text about what to expect to be written in each one of these files. For example, I can infer that the project file [.PRM] stores all the project settings, however, for the file CompEC.OUT I cannot make any inference if I'm not an experienced user of AquaCROP. The same reasoning applies to the outputs of the "Plug-in".

Response: We have added more information about

- the differences of the standard GUI and plug-in versions (Line 116 - 121)
- information on each of the output files from the standard GUI and plug-in versions (Line 190-222)
- details of output files and variables are also listed in a table provided as supplementary material Table S1.

- Comment 5: In the Step 4: Analysis, the authors provide an explanation that "AquaCropPlotter offers two simple statistical analysis options for gaining more insights into the data". The first one is a window summary function that is not clear to me what it is exactly. The authors are encouraged to give more details about how the summary and trend of variables are computed in the application. Moreover, they should present one example for each analysis in the case study.

Response: We have added more information about the analysis functions (Line 276-292) and include an example in the case study (Line 338-344, 362-365, 380-383).

**Reviewer #2:** The manuscript is very well written and is a substantial improvement as an option to the traditional AquaCrop model software. However, the figures need to be improved because they are of poor quality (blurry).

Response: The figures seem to be blurry when the submission system auto-generates the pdf file of the manuscript for reviewers. We have double checked our figure files and make sure the final version uploaded is not blurry.
